# Popularity and Novelty Dynamics in Evolving Networks

**DOI:** 10.1038/s41598-018-24456-2

**Published:** 2018-04-20

**Authors:** Khushnood Abbas, Mingsheng Shang, Alireza Abbasi, Xin Luo, Jian Jun Xu, Yu-Xia Zhang

**Affiliations:** 1 Web Science Center, University of Electronic Science and Technology of China, Chengdu, China; 2 Chongqing Institute of Green and Intelligent Technology, Chinese Academy of Sciences, Chongqing, China; 3 School of Engineering and IT, The University of New South Wales (UNSW Australia), Canberra, Australia; 4 Physics and Photoelectricity School, South China University of Technology, Guangzhou, 510640 China

## Abstract

Network science plays a big role in the representation of real-world phenomena such as user-item bipartite networks presented in e-commerce or social media platforms. It provides researchers with tools and techniques to solve complex real-world problems. Identifying and predicting future popularity and importance of items in e-commerce or social media platform is a challenging task. Some items gain popularity repeatedly over time while some become popular and novel only once. This work aims to identify the key-factors: popularity and novelty. To do so, we consider two types of novelty predictions: items appearing in the popular ranking list for the first time; and items which were not in the popular list in the past time window, but might have been popular before the recent past time window. In order to identify the popular items, a careful consideration of macro-level analysis is needed. In this work we propose a model, which exploits item level information over a span of time to rank the importance of the item. We considered ageing or decay effect along with the recent link-gain of the items. We test our proposed model on four various real-world datasets using four information retrieval based metrics.

## Introduction

Online social networking sites and social media platforms enable people to communicate and share different forms of contents or items such as texts, web links, photos and videos. These create a huge amount of data on the interaction between online items and users. Understanding the behaviour of a user in a friendship network in Facebook and/or a following-follower relationship in Twitter or a movie in a movie rating platform such as Netflix is important in marketing and recommendation systems. Network science theories and graph-theoretic frameworks have been successful in solving many real-world problems in industry, social, natural and medical sciences such as information overload problems^1,2^. The approaches of network science can be used to hypothesis and analyze the relationship ‘among the users or items’ (mono-partite network) and the relationship ‘between users and items’ (bipartite network). These representations of networks are useful in prediction and modelling link formation and network dynamics, which outline how social media items (e.g., news, blog, post, videos, and application downloads, topics in discussion forums and product reviews) are adopted and influenced by their creators.

Even if one has detailed information about the items and the users who share them, it can still be incredibly challenging to predict which item will be popular in future among users^3,4^. Item popularity is found to be affected by the following features: ‘structure’; ‘content’; ‘early adopters’; and ‘temporal’ feature. It is arguable whether ‘content features’ is useful for the popularity prediction of items. Some researchers^4,5^ have found ‘content features’ is not useful while others have found it is^6^. Furthermore, it is found that along with the item features, the underlying network ‘structural features’ such as the number of followers of seed users in Twitter^6,7^ and Facebook^5^ is useful in predicting their popularity. It is discussed how popularity of online items exhibit temporal dynamics^8–10^. Among all the features, the ‘temporal features’ is considered as one of the best features for popularity prediction^4,11^. For example, ‘temporal features’ of early adoption of news articles on Digg (e.g., the number of likes news received during initial one hour) has shown to play an important role in future popularity prediction of online news articles^12^. It is easy to get the ‘temporal features’ and also they are independent of the item level or network level features. Therefore, models based on ‘temporal features’ are applicable in more applications. A solely temporal feature based models, are applied widely in a variety of areas such as Twitter^7,13^, citation count^14,15^ and the occurrence of earthquake^16^. Because of its generic nature, and that it avoids the cost of feature engineering for prediction, it is also applied in investigating the diffusion of items.

Due to the competition and fitness of the items, not all of them become popular, and only some retain their popularity. In the presence of the information overload problem, identifying these popular and novel items are needed from every aspect of life. It affects every area of daily life such as what item to consume, outcome of election, political discourse, community formation and many more. Web is being used these days for propagating information for their social, informational and consumer needs through vast social networks that extends far beyond the personal relation or even geography. Therefore social network is also playing an important role in dissemination of ideas, purchases and reputations. As people are more affected by their own social networks, therefore, research for novelty as well as popularity in social networks are also an important task among researchers. A few people would view or consume stale information. This is the reason most of the news aggregators, Twitter and Facebook order the content according to newness (novelty) of the item. A very important factor in allocation of user attention is the finite number of items that a user can attend from a recommendation list. In consequence, only top popular items are consumed even though there are potential novel items at the bottom of the list and consequently ends up to skewed popularity distribution^17,18^. This research presents a model which identifies these potential novel items without any cost of predicting already popular items.

## Results

The popularity growth of social media items is generally driven by three factors: the ‘preferential attachment’, the ‘aging’ phenomena and the ‘recent popularity’ of an item. To model ‘preferential attachment’ and ‘recent popularity’, we propose to use a parametric model which interpolates between total popularity and recent popularity of the item (node) for different parameter values. To consider ‘aging’ effect, we assume that every link ’s future influence decays exponentially. Finally, we combine both phenomena and present a mathematical model and test it using four information retrieval based metrics. We have considered four different data sets namely; Movielens, Facebook, Netflix and re-tweet data sets. In the case of re-tweet data, it has information about evolution of every tweet from zero (time) seconds. While in other cases, we have also information about the inception of the item in the system, i.e. we have a time line {*t*_0_, *t*_1_, …, *t*_*n*_, …} and every item introduced at a particular time say *t*_*n*_. In the case of re-tweet data birth time *t*_*n*_ = *t*_0_ for all the tweets while in other cases *t*_*n*_ can be any time in the system. Therefore, learning and prediction problem changes for re-tweet data set as compare to other data sets. Considering these factors, there are two types of the prediction problems: (1) From a given link formation temporal details for a given network at times *t*_*n*_, we need to predict the ranking of nodes after a future time window *T*_*F*_, according to: (a) link gain during a future time window *T*_*F*_, or (b) total link gain up to the future time window *T*_*F*_ (this case is only applicable for re-tweet data for Reinforced Poisson Process Model^13^(RPPM) model testing, due to the nature of the data and model prediction, see Method section for information about data and model). In our model, we consider the bipartite network which consists of a set of users (*U*) and a set of objects (*O*), as online items. If a user *u* (*u* ∈ *U* collect the object *o* (*o* ∈ *O*), then there is a link from *u* to *o*. Our prediction model ranks objects or nodes according to their number of links they will receive during the future time window *T*_*F*_. Further we take 10 random times {*t*_1_, *t*_2_, …*t*_*r*_, …, *t*_10_}, which are selected from the middle one third of the time sequence so that there are enough history and future information for most of the items. After evaluating the result on the metrics (Precision, Novelty, *AUC*, and Temporal Novelty) for each random time, we take the average of the results. In calculating accuracy, we only consider those items which have received at least one link before a random time. Then we test our model based on real link gain during the future time window *T*_*F*_. (2). In second type of prediction problem for re-tweet data set, we split the data according to some time *t*_*k*_ and we make the prediction for every re-tweet after time *t*. In this case, prediction problem is to rank on the basis of the total (absolute) number of link gain for every tweet at time *t* + *T*_*F*_.

In parameter learning, the parameters *λ* and *γ* in Eq. 9 are accepted, which maximize the precision during 3000 iterations. Only in the case of re-tweet data, the learned parameter is different for every individual retweet. In other cases, we took an average of the parameter values for all the items, as the nature of the data does not support learning for individual items. Furthermore, in this study we compare the performance of the proposed model to three well-known models (Popularity Based Predictor^19^ (PBP), Degree (Preferential Attachment) and Reinforced Poisson Process Model (RPPM)) by analyzing the sensitivity of the models. Since RPPM learns the parameter from initial adoption history of items so the re-tweet data are used to test its performance.

### Results for varying top *k* list size

**Figure 1:**
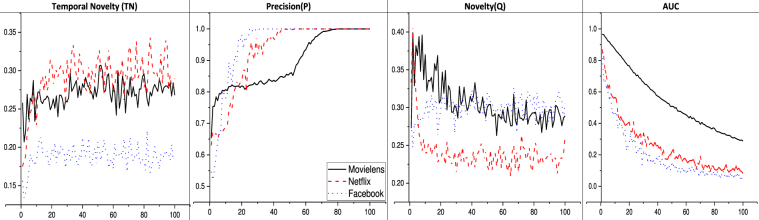
Model performance results for varying top *k* size of the popular list. The X-axis shows the percentage of the total list size as top *k* number. Y-axis shows the performance index considering Temporal Novelty (*TN*), Precision (*P*), Novelty (*Q*) and *AUC* for top 100 items; all indices lies between 0 and 1, the higher the better. The black solid line is the results for Movielens, the red line with dashes is for Netflix, and the blue dotted line is for Facebook.

In order to get and compare the accuracy results for varying size of top k items in the popular list (shown in Fig. 1), we have used the following four information retrieval metrics: (a) Novelty (Q): quantifies the objects which enter in top popularity list for the first time (an absolute novelty); (2) Temporal Novelty (TN): reflects the ability to predict the objects which did not gain popularity in the past time window but they appear in the top popular list in future; (3) Precision (P): the fraction of correctly predicted objects using the top 100 popular objects; and (4) Area Under receiving operating Characteristic (AUC): gives the comparative ranking ability of the predictor. TN and Q metrics are very sensitive as it depends on exact identification of items which where not available in past or recent past time window. Considering temporal novelty (TN, Eq. 16) as an accuracy metric, with respect to top k list size, the proposed model outperforms in the case of Netflix (see Data and Metrics section for detailed data description) than the rest. For precision (P, Eq. 12) analysis, the accuracy increases with different rates for different datasets, most likely due to different nature of the generated datasets. Therefore, it is better to use larger *k* (30%+ for Facebook, 50%+ for Netflix, and 70%+ for Movielens) to get 100% precision. In the case of novelty (Q, Eq. 13) analysis, the accuracy remains constant as list size increases. In the case of AUC, performance decreases with the size of the list; all decreasing with a similar trend.

### Varying both past and future time windows with equal value (varying *T*_*P*_ = *T*_*F*_)

**Figure 2:**
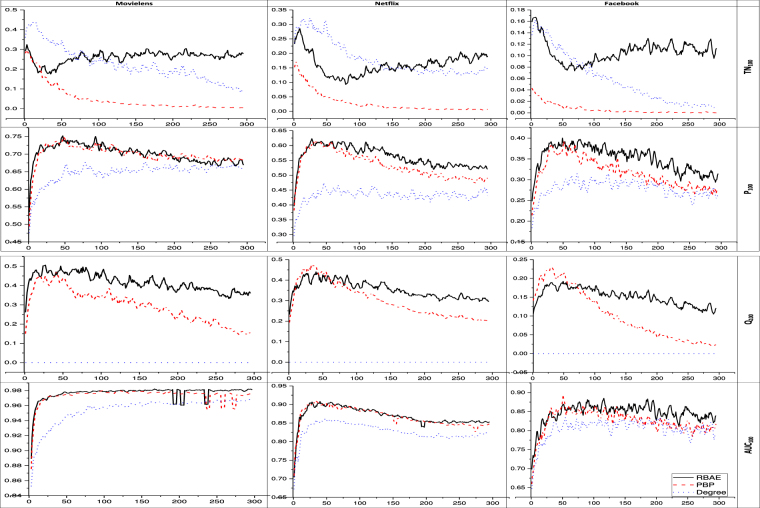
Results for varying future and past time windows for top 100 items in the popular list. The X-axis is the number of time tick up to 300 days considering equal past and the future time windows. Y-axis is the performance values. All metrics lie between 0 and 1, higher the better. The dotted blue line is for degree, the red line with dashes is for PBP, and solid black line is for RBAE.

To test the model’s ability to make a correct prediction, it is compared to the benchmark models, for varying past and future time windows (*T*_*P*_ and *T*_*F*_) but having equal values, using the four information retrieval based indices considering only top 100 items of the popular list (*k* = 100). Based on the results depicted in Fig. 2, on average the performance of the proposed model, Recent Behaviour with Aging Effect (RBAE), is better than the other two benchmark models as they have either ability to predict in only one case such as in the case of Temporal Novelty (*TN*). Novelty (*Q*) index performs better than RBAE for initial few days of prediction degree but after few days RBAE outperforms all. As shown in Fig. 2 for the top 100 popular items, Temporal Novelty (*TN*_100_) values increase as the past and future time windows increase for values above 100 days for all the datasets. Overall, RBAE model outperforms both benchmark models as time windows increases. Considering Precision (*P*_100_), RBAE model outperforms the other two models in Netflix and Facebook and has similar performance with PBP for Movielens dataset, despite a slight decreasing trend as the time window increases. Novelty (*Q*_100_) or absolute novelty (Eq. 13) results show that our model outperforms other two models in Movielens and after around 75 days in the other two datasets. Considering *AUC*_100_, as shown in Fig. 2, RBAE model performance is always better (or equal to PBP) in all the datasets and for all the time windows.

### Varying future time window (*T*_*F*_)

**Figure 3:**
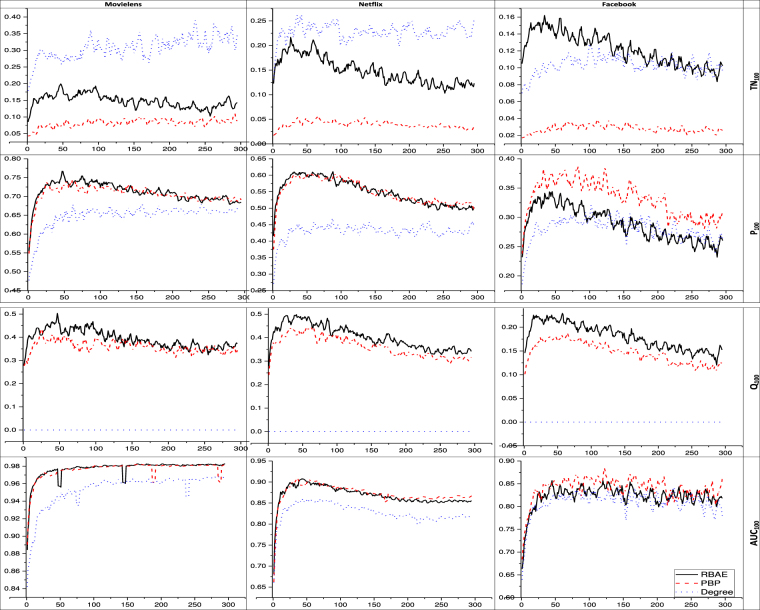
Results for different values of future time window *T*_*F*_. Y-axis is the value for performance measures for the top 100 popular items. All indices lie between 0 and 1, the higher the better. The dotted blue line is for degree, the red line with dashes is for PBP, and solid black line is for RBAE.

Figure 3 depicts the performance of proposed predictor against the benchmark predictors for different values of the future time window up to 300 days. Similar to author^19^, the past time window length *T*_*P*_ = 60 days is considered. For proposed predictor (RBAE), the parameter learned as described in Method section. For PBP the parameter values are iterated up to two decimal places and chose which gave the best precision. As the results of the analysis based on the four performance indicators presented in Fig. 3 shows, on average RBAE outperforms the benchmark models. For example, the ability of degree in making a prediction for temporal novelty (*TN*_100_) is best while it shows zero performance in the case of absolute novelty (*Q*_100_). PBP performs better than Degree but RBAE performs consistently better in all the cases. As the results of the analysis for Temporal Novelty (*TN*_100_) shows our proposed model, RBAE, always performs better than PBP; degree performs better than RBAE in Movielens and Netflix datasets while in the case of Facebook data, RBAE outperforms both benchmark models. Precision(*P*_100_) results reflects RBAE performs better than degree in all cases and being almost similar accuracy to PBP for Movielens and Netflix datasets while in the case of Facebook PBP outperforms RBAE. The results of novelty (*Q*_100_) analysis show that RBAE performs better than both benchmark models in all the cases. It is also important to note that novelty affected by future time window size.

### Predicting the absolute popularity

**Figure 4:**
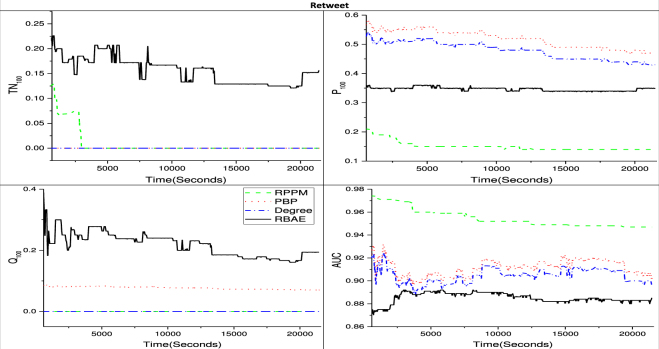
Results for different values on future time window *T*_*F*_. The X-axis shows the time (*T*_*F*_) in seconds. On Y-axis we depicted the index used for performance measured i.e. Precision(*P*_100_), Temporal Novelty(*TN*_100_), Novelty (*Q*_100_), and *AUC*_100_ for top 100 items. All metrics lie between 0 and 1, the higher the better. The solid black line is for RBAE, the red dotted line for PBP, blue line with dash and dots is for Degree and green line with dashes is for RPPM.

In this section, we compare the proposed model, RBAE, with the Reinforced Poisson Process Model (RPPM) model, which is for predicting absolute number of popularity gain, in addition to the other two benchmark models (Degree and PBP) considering the total number of link gains up to a future time window. Twitter re-tweet data is used. To make prediction the model is trained for 20 minutes by considering recent past time window for 10 minutes (*T*_*P*_ = 600 seconds). As shown in Fig. 4, At every time step in future, the total number of re-shares is counted and the tweets are ranked accordingly. It is found that in the cases of temporal novelty (*TN*) and novelty (*Q*), RBAE prediction outperforms other models while in the other cases its performance is not good.

## Discussion

This study attempts to solve the problem of predicting popularity of potential items^18^ which are generally suppressed by already popular items. We solve this problem by considering user-item bipartite interaction network and ranking approach. We emphasize two kinds of novelty prediction: ‘absolute novelty’ and ‘temporal novelty’. From Fig. 1, we find that as ranking list size increases, precision also increases, *AUC* decreases, while the novelty and temporal novelty are slightly affected. This result shows our model performs well only for ranking top popular items. It also suggests discovering novel items has cost of accurately predicting lower rank items. The similar result is also found from Fig. 4, as RBAE outperforms other models in predicting novelty and temporal novelty but not in other two metrics. From Fig. 3, we can say the long-term prediction performance increases with recent past time window size. This suggests our model is sensitive towards recent past window size selection on all the datasets. In Fig. 3 we also see the effect of fixed recent past time window for varying future time window, RBAE performs for Movielens and Netflix dataset but in the other cases its performance is equal or it outperforms. This analysis suggests recent past time window affect more in identifying items which did not get popularity during recent past time window. Further it is found that proposed predictor does not perform well for Facebook system on precision metric as compared to PBP when the past time window is fixed (see Fig. 3), but in other cases, it is found that it makes good prediction when the past time window is also varying (see Fig. 2 for same Facebook system). Thus we can say that RBAE is an optimal predictor because it helps in predicting and ranking novel items. From Fig. 4, a limitation of our proposed model is that it does not perform well for ranking on the basis of total popularity gain (see problem definition 2) as AUC and precision is vital metrics. Nevertheless, RBAE outperforms the other models in predicting both novel as well as temporal novel items. The proposed predictor is purely temporal feature based, which is also found to be effective in generalization^4^. We have performed extensive experiments on four distinct data sets, which represent four distinct systems. Our model can also be applied to other evolving systems. For future possible work, we will consider the temporal features along with other driving factors such as preferential attachment, aging, freshness of item, community, non-linear preferential attachment, and sentiment analysis.

## Methods

We first describe three benchmark models, and then we introduce our proposed model. The benchmark models are given as follows

### Degree

Matthew effect or preferential attachment is a well-known phenomenon which is seen almost in every evolving network. It states the rate of a node’s future link gain (e.g., movies receiving new rating in the case of Movielens, friends receiving new likes or comments in the case of Facebook wall post activities) is proportional to the number of links it currently has. In other words, the current degree of an item (*k*_*o*_ (*t*)) is a good predictor for its future popularity.

### Popularity-based predictor

PBP, proposed by^19^, extends the degree (or preferential attachment) model by adding a new parameter, ‘recent time window’, as a proxy for items’ recent popularity. The prediction score of an item at time *t* can be given as:

1 $${{\rm{s}}}_{o}{(t,T}_{{\rm{p}}})={{\rm{k}}}_{o}(t)-\lambda {{\rm{k}}}_{o}(t-{{\rm{T}}}_{{\rm{P}}}),$$

where s_*o*_(t, T_P_) is the predicted rating/links considering recent (past) time window *T*_*P*_ from t. k_*o*_(t) is the total link gain up to time t. *λ* ∈ [0, 1] and *λ* = 0 gives the total popularity (i.e., the total number of links for an item) and for *λ* = 1 gives recent popularity (i.e., the number of links in recent time window *T*_*P*_).

### Reinforced Poisson Process Model

RPPM is proposed by^13–15^ for predicting popularity dynamics of evolving systems. Consider time-dependent Poisson process which gives the intensity of a given message (*m*), its popularity (re-tweet) dynamics $\{{t}_{k}^{m}\}$ up to time *T*_*i*_, can be modelled as reinforced Poisson process with intensity *λ*_*m*_(*t*, *k*) which can be measured as

2 $${\lambda }_{m}(t,k)={c}_{m}{f}_{m}(t){r}_{m}(k),$$

where *c*_*m*_ is the intrinsic attractiveness ${f}_{m}({t}_{k})={t}_{k}^{\gamma }$ is the time relaxation function which characterize aging effect. *r*_*m*_(*k*) is the reinforcement function depicting the “rich-gets-richer” effect. Further they modeled reinforcement mechanism as follows-

3 $${r}_{m}(k)=\,\in \,+\frac{(1-{e}^{-\alpha (k+1)})}{(1-{e}^{\alpha })},$$

where *r*_*m*_(*k*) is reinforcement mechanism and *k* is cumulative number of re-tweet at time *t*. The model parameters {*c*_*m*_, *α*_*m*_, *γ*_*m*_} is estimated by maximizing the likelihood function^13^. The cumulative number of retweet count at any time in future *t* can be estimated by expectation of Poisson process,

4 $$\frac{dR}{dt}=\lambda (t),$$

which can be solved exactly as following expression with boundary condition *R*(*T*_*i*_) = *n*.

5 $$R(t)=\frac{({ln}(1+{e}^{Y})-Y-\,{ln}\,{\varepsilon }-{\alpha }^{\ast })}{{\alpha }^{\ast }},$$

where,

6 $$Y={\varepsilon }{c}^{\ast }{\alpha }^{\ast }\frac{({T}_{i}^{1-{\gamma }^{\ast }}-{t}^{1-{\gamma }^{\ast }})}{\mathrm{(1}-{\gamma }^{\ast }\mathrm{)(1}-e-{\alpha }^{\ast })}-(n+\mathrm{1)}{\alpha }^{\ast }-ln({\varepsilon }-e-{\alpha }^{\ast }(n+\mathrm{1)),}$$

where, {c*, α*, γ*} is the estimated parameter after likelihood maximization, and *ε* = 1 + ∈ (1 − *e*^−*α*^).

### Our proposed model: Considering aging factor with recent popularity

The popularity of a node in a complex system is driven by four factors: its degree, newness^20^, recent popularity gain^21^ and aging effect^15,22–24^. When the number of nodes in a system is very large we assume that attraction of attention due to newness is negligible. To consider recent popularity and degree together, we consider a parametric linear model which uses total popularity and recent popularity. The recent popularity is also used in previous research^19,21^. Since in an ideal rich-gets-richer system oldest node is the popular one and therefore recent popularity gain should also be a good predictor. But since the Web system are driven by many intrinsic as well as extrinsic phenomena^25–28^ therefore we have kept it parametric. As aging phenomenon is omnipresent in many complex systems so in web system also, for example in social media platforms, microblogs lose their popularity^13^, pathogenes lose their infectiousness due to ageing^24^ and network changes structure due to the ageing factor over time^29^. Modeling of aging phenomenon depends on system such as be exponential^22,23,30^, power-law^7,13,31^ and lognormal^14,15^. In our study we have considered exponential decay effect. To consider all these facts, we come up with an intuitive solution that aging factor with recent popularity will help us in detecting “potential items” (going to be popular). If s_*o*_(t, T_p_) is prediction score at time *t* given the past time window *T*_*P*_. We can say

7 $${s}_{o}(t,{T}_{p}) \sim \,\frac{({k}_{o}(t)-\lambda {k}_{o}(t-{T}_{P}))}{\sum _{O}({k}_{o}(t)-\lambda {k}_{o}(t-{T}_{P}))}$$

The above equation states that score of the object follows its recent popularity gain. *λ* is tunable parameter between recentness and total popularity. It can take values in [0, 1] interval. As the ageing or decay is present everywhere, so we can formulate the prediction score as follows

8 $${s}_{o}(t,{T}_{p}) \sim \,\frac{\sum _{u}{e}^{\gamma ({T}_{uo}-t)}}{\sum _{O}\sum _{u}{e}^{\gamma ({T}_{uo}-t)}}$$

where *T*_*uo*_ denotes the time at which user *u* consumed the object *o* and *γ* is free parameter. Since recent popularity will be good predictor if decay rate is constant, therefore, we will have

9 $${s}_{o}(t,{T}_{p}) \sim \,\frac{\sum _{u}{e}^{\gamma ({T}_{uo}-t)}}{\sum _{O}\sum _{u}{e}^{\gamma ({T}_{uo}-t)}}\bullet \frac{({k}_{o}(t)-\lambda {k}_{o}(t-{T}_{P}))}{\sum _{O}({k}_{o}(t)-\lambda {k}_{o}(t-{T}_{P}))}$$

In the above model, in the case of monopartite networks user *u* is the set of other nodes from where node or object *o* have received the link. For ease of representation, we name it as Recent Behaviour with Aging Effect (RBAE).

### Parameter learning using gradient descent

To optimise the model parameters we use gradient descent method and apply the following two cost minimization approaches:

- *Ordinal ranking minimization*, in which we first rank the predicted and real values and then the learned the parameters.
- *Normalised score minimization*, in which we normalise the both predicted and real scores between 0 and 1 and then learn the parameters. Further, we apply a weight to the cost by 1 − *P*_*n*_ and 1 − *Q*_*n*_.

For learning the parameters in our proposed model (9) we use gradient descent and we have calculated the gradients as

10 $$\begin{array}{rcl}\frac{\partial ({s}_{o}(t,{T}_{P}))}{\partial \lambda } & = & \frac{[(({k}_{o}(t)-\lambda {k}_{o}(t-{T}_{P})).(\sum _{o}({k}_{o}(t-{T}_{P}))))-(({k}_{o}(t-{T}_{P}))(\sum _{o}({k}_{o}(t)-\lambda {k}_{o}(t-{T}_{P}))))]}{{(\sum _{o}({k}_{o}(t)-\lambda {k}_{o}(t-{T}_{P})))}^{2}}\\  &  & .(\frac{\sum _{u}{e}^{\gamma ({T}_{uo}-t)}}{\sum _{o}\sum _{u}{e}^{\gamma ({T}_{uo}-t)}}),\end{array}$$

11 $$\begin{array}{rcl}\frac{\partial ({s}_{o}(t,{T}_{P}))}{\partial \gamma } & = & (\frac{({k}_{o}(t)-\lambda {k}_{o}(t-{T}_{P}))}{\sum _{o}({k}_{o}(t)-\lambda {k}_{o}(t-{T}_{P}))})\\  &  & .(\frac{[(\sum _{u}{e}^{\gamma ({T}_{uo}-t)}.({T}_{uo}-t)).(\sum _{o}\sum _{u}{e}^{\gamma ({T}_{uo}-t)})]-[(\sum _{u}{e}^{\gamma ({T}_{uo}-t)}).(\sum _{o}\sum _{u}{e}^{\gamma ({T}_{uo}-t)}({T}_{uo}-t))]}{{(\sum _{o}\sum _{u}{e}^{\gamma ({T}_{uo}-t)})}^{2}}),\end{array}$$

So we updated parameter as follows:-

$$\begin{array}{rcl}{\lambda }_{i} & = & {\lambda }_{i}-\alpha \mathrm{.(}{\rm{\Delta }}e\mathrm{).}(\frac{\partial {s}_{o}(t,{T}_{P})}{\partial {\lambda }_{i}}),\\ {\gamma }_{i} & = & {\gamma }_{i}-\alpha \mathrm{.(}{\rm{\Delta }}e\mathrm{).}(\frac{\partial {s}_{o}(t,{T}_{P})}{\partial {\gamma }_{i}}),\end{array}$$

where parameters *λ* and *γ* are the same as in Eq. 9 and Δ*e* is the error magnitude which can be calculated considering different scenarios such as ordinal ranking-based, and normalised score error minimization. Since we want to maximize accuracy while learning, we give the weight of 1 − *P*_*n*_ to normalised score based on the error minimization in our current result. We also test the result considering normalised score minimization approach and found it is also working good; we accepted the parameters which give the best accuracy. While parameter estimation, we set the past and future time window as 45 days, in the case of Movielens, Netflix and Facebook. In the case of Twitter, we learn the parameter for initial 20 minutes of re-sharing data and kept past time window for 10 minutes.

## Data and Metrics

To test the performance and robustness of our model, we consider the following datasets and evaluation metrics:

### Data

To test the predictor’s accuracy we have used different data sets. Like MovieLens, Netflix, Facebook wall post and retweet data from Twitter set-

- **Netflix**: This data set contains movie ratings from a famous platform called Netflix. The original dataset has 480, 189 users, 17, 770 items and 100, 480,507 ratings between 1 January 2000 and 31 December 2005. It contains rating from 1 to 5, where 1 being the worst and 5 is the best. We have randomly selected user’s who have rated at least 10 movies above 2.
- **Movielens 10** **M**: This dataset contains record of the movie ratings by users during 01 January, 2002 to 1^*st*^ January 2005. MovieLens is provided by orgGroupLens project at University of Minnesota and contains 10, 000, 054 ratings and 95, 580 tags applied to 10681 movies by, 71567 users of the online movie recommender service MovieLens^32^. It contains rating from 1 to 5 where 1 is the worst and 5 is the best. We only consider positive ratings, where there is a link between a user if he/she has rated a movie higher than 2. We have randomly sampled 7, 000 unique users and all the movies rated by them. Further, we used the day as a unit of time rather than the detailed time.
- **Facebook wall post**: This dataset contains user’s wall post activity information during 14 October 2004 to 21 January 2009. It contains 46, 951 users and their wall post activity^33,34^. We ignored the self-influence, i.e. the record where the user has acted on his own wall. Further, we have converted this into a bipartite network where there is a link between a users and a Facebook wall when the user post a content to another user’s wall.
- **Twitter re-tweet Data**: This dataset contains tweet and re-tweet information^7^ on Twitter site. The original data contains 3.2 billion tweets and re-tweets on Twitter from 7 October to 7 November 2011. In our study, we randomly sampled 5000 tweets and all the information about their re-tweet activity. The re-tweet time is taken as relative, which is the main difference between this data and other data set used in this study. Every tweet has assigned time as 0 second when it was first shared. The time is considered in seconds.

The data description after cleaning are as in Table 1. In the table number of user for re-tweet data is dummy. Since in the data the user detail is not available so we consider every retweet or like is coming from different user therefore the details in the table is maximum possible user for Re-tweet data set.

**Table 1:** Information about the processed data.

Data set	Users	Objects	Links
Netflix	4960	16599	1.2 × 10^6^
Movielens	9466	861775	1.2 × 10^6^
Facebook	40981	38143	8.6 × 10^5^
Re-tweet	—	5000	1.06 × 10^6^

### Evaluation metrics

The following evaluation metrics are adopted to measure the accuracy of the proposed models:*Precision* (*P*_*k*_), *Novelty* (*Q*_*k*_), *Temporal Novelty(TN*_*k*_) and *Area Under receiving operating Characteristic*(*AUC*_*K*_), also referred as ROC^35^.

- *Precision* is defined as the fraction of objects listed in the top *k* rankings of the predicted and real ranking lists^36^,
  12 $${P}_{k}=\frac{{D}_{k}}{k},$$
  where *D*_*k*_ is the number of common objects in the top *k* of both predicted and real ranking lists. *P*_*k*_ ∈ [0, 1]. The higher value of *P*_*k*_, the better precision of prediction.
- *Novelty(Q*_*k*_) measures the ability of a predictor to rank ‘new object’ in the top *k* position that was not in top *k* position in past. Let *R*_*k*_ denote the number of new objects (that were not in top rank before) in the top *k* of the real list. And *E*_*K*_ denotes the number of the new objects correctly predicted by our model in the top *k* ranking list. Then the novelty score is given by
  13 $${Q}_{k}=\frac{{E}_{k}}{{R}_{k}},$$
- *AUC* measures the importance of the relative position of its top *k* objectives in the predicted and ranked list. It selects top *k* objects from the real list as a benchmark and compares its rank score in top *k* predicted list. Let *s*_*p*_ ∈ *L*_*p*_ and *s*_*r*_ ∈ *L*_*r*_ be the scores of an object in predicted list. Then *AUC* is given by
  14 $$AUC=\frac{\sum _{{s}_{p}\,\in \,{L}_{p}}\sum _{{s}_{r}\,\in \,{L}_{r}}I({s}_{p},{s}_{r})}{|{L}_{p}||{L}_{r}|}$$
  *where*,
  15 $$\begin{array}{rcl}I({s}_{p},{s}_{r}) & = & \{\begin{array}{ccc}0, & \,{\rm{if}} & {s}_{p} > {s}_{r},\\ 0.5, & \,{\rm{if}} & {s}_{p}={s}_{r},\\ 1, & \,{\rm{if}} & {s}_{p} < {s}_{r}.\end{array}\end{array}$$
- *Temporal Novelty(TN*_*k*_) measures the ability of a predictor to rank ‘new object’ in top *k* that was not present in the top *k* position during recent past time window but during future time window *T*_*F*_ they gained popularity. Let ${R}_{k}^{{\rm{\Delta }}t}$ denote the number of new objects (that were not in top rank by popularity gain during recent time window *T*_*P*_) in top *k* of the real list. And ${E}_{k}^{{\rm{\Delta }}t}$ denotes the number of the new objects correctly predicted by our model in the top *k* ranking list. Then the temporal novelty (*TN*_*k*_) score is given by

16 $$T{N}_{k}=\frac{{E}_{k}^{{\rm{\Delta }}t}}{{R}_{k}^{{\rm{\Delta }}t}},$$
